# Emerging roles of the cancerous inhibitor of protein phosphatase 2A (CIP2A) in ovarian cancer

**DOI:** 10.1038/s41598-025-05013-0

**Published:** 2025-07-01

**Authors:** Alice Filipe, Sayeh Saravi, Denis Mustafov, Suzana Panfilov, Simran Banger, Seyedehnajmeh Mousavikivaj, Maria Braoudaki, Senthilkumar Kailasam, Yasser Riazalhosseini, Michelle A. Sahai, Fotios Drenos, Cristina Sisu, Emmanouil Karteris

**Affiliations:** 1 Department of Biosciences, College of Health, Medicine and Life Sciences, Brunel University of London, Uxbridge, UB8 3PH UK; 2 School of Life and Medical Sciences, University of Hertfordshire, Hatfield, AL10 9JA UK; 3 Victor Phillip Dahdaleh Institute of Genomic Medicine at McGill University, Montreal, QC H3A 0G4 Canada

**Keywords:** Cancer, Oncology

## Abstract

Ovarian cancer (OvCa) is the sixth most common gynaecological cancer in the UK, accounting for over 200,000 deaths worldwide. Cancerous Inhibitor of Phosphatase 2 A (CIP2A) is an oncoprotein and an endogenous inhibitor of PP2A. CIP2A is a key regulator for cellular processes (e.g. proliferation, DNA damage) and is involved in the progression of many malignancies. In this study we provide a comprehensive overview of its role in OvCa making use of in silico tools, clinical samples and in vitro models. CIP2A is overexpressed in OvCa patients, with metastatic patients having significantly higher expression when compared to patients with malignant and benign ovarian tumours. High CIP2A expression reduces both overall-and progression-free survival, whereas an R530T mutation is predicted to cause structural destabilisation of the CIP2A dimer. We also provide evidence for microRNA (miRNA) and mRNA target interactions with *CIP2A*. Finally, we have studied the effects of CIP2A inhibition in an in vitro BRCA2 model compared to BRCA2 wild-type OvCa cells, using RNA-sequencing. Gene enrichment pointed towards changes p53 pathway, protein metabolism, transporter activity, DNA replication, and cell cycle. Our data provide a novel insight into the role of CIP2A in OvCa and the potential of drug repurposing for therapeutic interventions.

**Supplementary Information:**

The online version contains supplementary material available at 10.1038/s41598-025-05013-0.

## Introduction

Ovarian cancer (OvCa) is one of the leading causes of death for female patients with gynaecological cancers, responsible for 313,959 cases worldwide annually^1^. OvCa is an umbrella term for an amalgamation of different ovarian malignancies, each of which presents with distinct features, affecting disease progression^2^. Due to a lack of effective screening^3^ as well as vague and confounding symptoms^4^approximately 70% of patients are diagnosed at the later stages (III or IV)^3,4^. Currently, no screening program for OvCa exists for the general population. This is because overall, the incidence of the disease is low and false positive screens for OvCa can result in unwanted interventions. Additionally, a screening test must detect OvCa in a state where it is treatable and potentially curable. Current strategies to diagnose OvCa are based primarily on blood tests (e.g. measuring CA-125, HE4) and imaging, both of which have certain limitations^5,6^.

The tumour suppressor, protein phosphatase 2 A (PP2A), is a serine/threonine phosphatase of vital importance for regulating many processes in the cell, such as cell growth, proliferation and apoptosis^7^. Cancerous inhibitor of PP2A (CIP2A) is a 90 kDa oncoprotein, encoded by the gene *KIAA1524* located at 3q13.13^8,9^. It functions as an endogenous PP2A inhibitor and plays a non-essential role in healthy physiology, since it has minimal expression in healthy tissues. Indeed, its highest expression has been documented in immune, intestinal and sperm cells^9^; contrary to its upregulation in certain cancers. These findings corroborate data from the Genotype-Tissue Expression (GTEx) portal, where very low expression of PP2A is measured in normal tissues including: adipose tissue, brain, breast, cervix, colon, fallopian tubes, heart, kidney, ovary, pancreas, prostate, stomach, uterus, vagina, and whole blood^10^.

Through inhibition of PP2A, CIP2A drives dysregulation of signalling pathways, since it can interact with other oncoproteins such as MYC, Akt, as well as mTOR to encourage tumour progression^11^. A number of studies have shown that *CIP2A* is over-expressed in many cancers including breast^12^,endometrial^13^and cervical^14^. In OvCa, *CIP2A* has been shown to be over-expressed in serous, endometrioid, mucinous, and clear cell carcinoma^15^ with increased cytoplasmic CIP2A expression being associated with higher grade, more aggressive disease and reduced overall survival (OS) and progression-free survival (PFS). Of note, *CIP2A* over-expression in OvCa, has been shown to correlate to chemotherapy resistance^16^.

In this study, we provide a deeper insight into the role of CIP2A in OvCa making use of in silico tools, RNA-sequencing, tissue microarrays and functional assays. More specifically, we studied the expression patterns of *CIP2A*, identified functional miRNA-mRNA target interactions (MTIs), modelled mutations involved in OvCa and studied its clinical utility as a biomarker. We have expanded on these observations, by mapping the protein distribution of CIP2A on a tissue microarray. Finally, we investigated the effect of CIP2A inhibition on the OvCa transcriptome in vitro, using TD-19, an Erlotinib derivative marketed as a CIP2A inhibitor.

## Results

### Expression of CIP2A (KIAA1524) in health and gynaecological malignancies

**Fig. 1:**
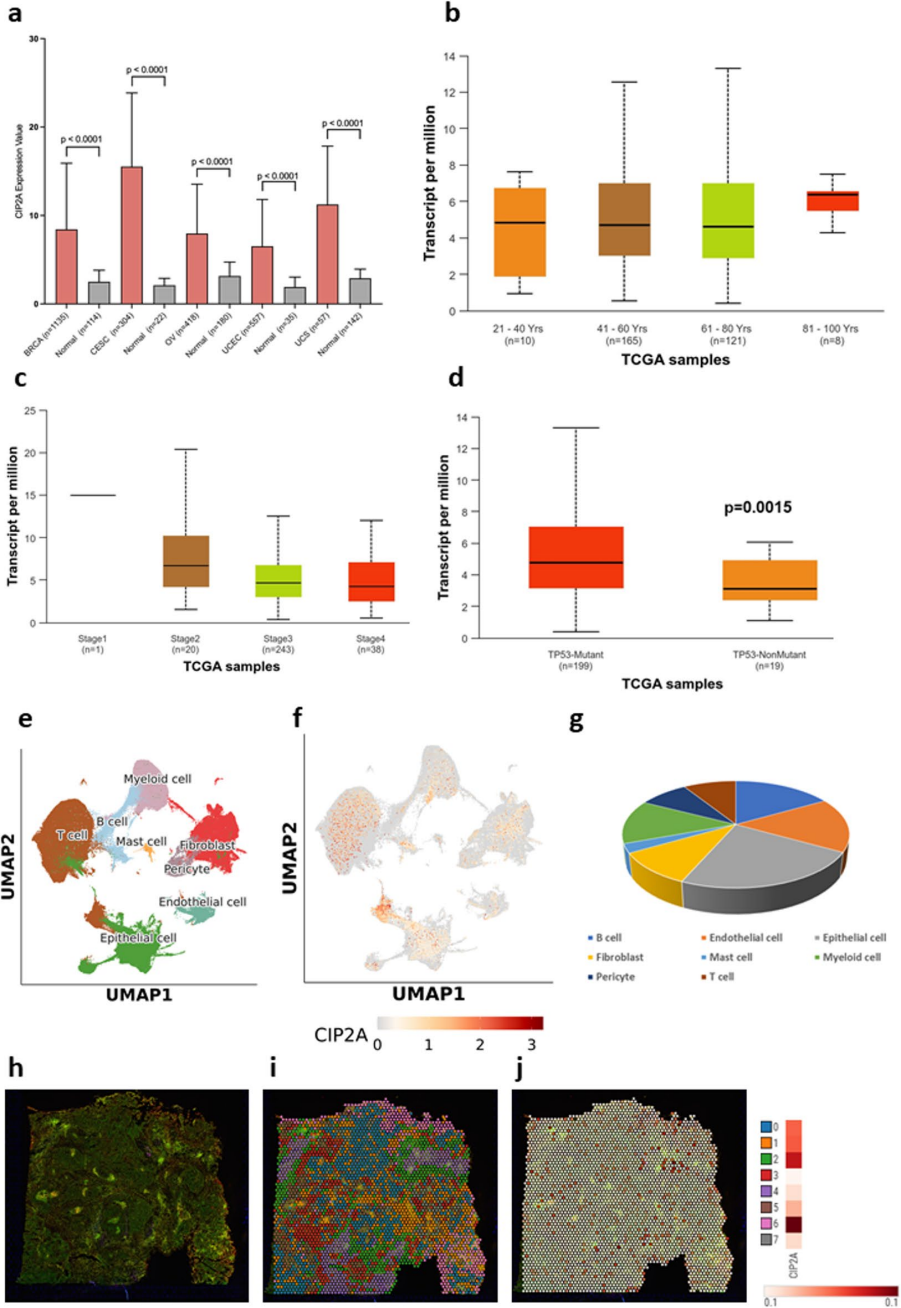
CIP2A (KIAA1524 ) is aberrantly expressed in ovarian cancer. *CIP2A* is significantly upregulated in breast invasive carcinoma (*BRCA*), cervical squamous cell carcinoma and endocervical adenocarcinoma (CESC), ovarian serous cystadenocarcinoma (OV), uterine corpus endometrial carcinoma (UCEC), and uterine carcinosarcoma (UCS), when compared to healthy controls **(a)**. *CIP2A* expression is not dependent on patients’ age (**b)**, stages of OvCa (**c)**, but is upregulated in OvCa patients that have TP53 mutations **(d)**. Ovarian T/B/mast/myeloid/epithelial/endothelial cells, pericytes and fibroblasts express *CIP2A* (**e-g**). Spatial transcriptomics data on the expression of CIP2A across eight different clusters of a single patient with endometrial adenocarcinoma of the ovary (**h-j**).

Data from the Genotype-Tissue Expression (GTEx) Project^10^ demonstrate that *CIP2A* is co-expressed with *PP2A* in female organs under normal conditions, demonstrating a weak positive correlation (Supplementary Fig. 1a-c). Leveraging GTEx and the Cancer Genome Atlas (TCGA) data^17^we demonstrate that *CIP2A* is significantly upregulated in various gynaecological malignancies, including OvCa (Fig. 1a), irrespective of age of patients (Fig. 1b) or stage (Fig. 1c), whereas *CIP2A* was upregulated in Grade 3 (i.e. poorly differentiated, high grade) when compared to Grade 2 (Supplementary Fig. 1d). Consistently with results from *CIP2A* breast cancer studies^18,19^OvCa patients who carry a TP53 mutation, have significantly higher levels of *CIP2A*, when compared to TP53-non-mutants (Fig. 1d; UALCAN^20^). Using an OvCa single cell database, of 84 ovarian tumour patients -primarily high-grade serous carcinoma (HGSC)- from 16 publicly available studies^21^we demonstrate that *CIP2A* is expressed in a wide range of cells i.e., T/B/mast/myeloid/ epithelial/endothelial cells, pericytes and fibroblasts (Figs. 1e-g). Aberrant *CIP2A* expression in OvCa was further corroborated using information from the Spatial Transcript Omics DataBase (STOmics DB). There was no difference in the expression of CIP2A across the different clusters of a single patient with endometrial adenocarcinoma of the ovary. This is an ovarian primary tumour with no evidence of cancer spread to regional lymph nodes, or distant metastasis (T1N0M0; Figs. 1h-j)^22^.

### CIP2A is not frequently mutated in OvCa

**Fig. 2:**
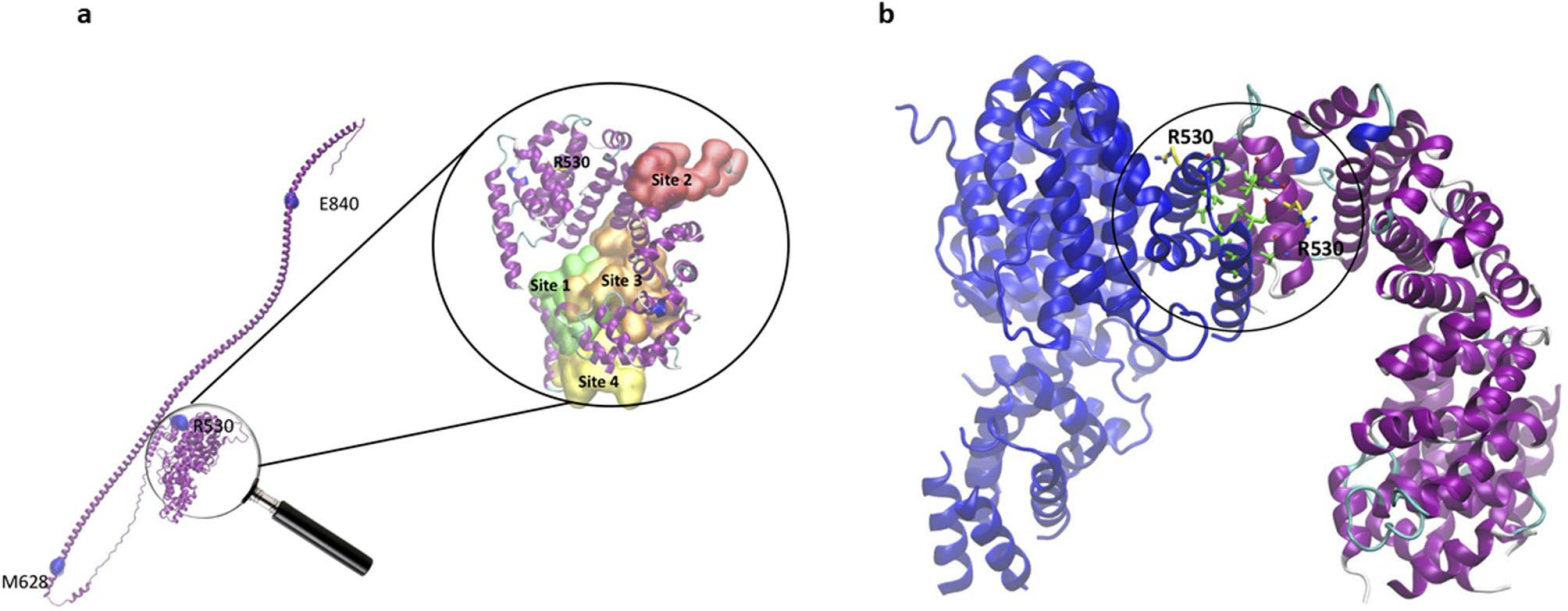
(**a**) Structure of the wild-type CIP2A protein as predicted by AlphaFold2 (Identifier: AF-Q8TCG1-F1) (purple). The three potential sites for the E840K, M628I, and R530T mutations are highlighted in blue spheres. The zoomed-in image shows the location of one of the mutation sites, R530 with respect to the four previously predicted ligand binding sites. (**b**) 3D structure of the apo-CIP2A dimer (PDB ID: 5UFL), revealing the dimerisation interface involving the residues L529, V525, L532, L533, L546, L550 (green) and R530 (yellow) between chain A (blue) and chain B (purple). The structures were processed and images generated using the VMD (Visual Molecular Dynamics) software.

The online tool cBioPortal^23^ was used to determine gene alterations of *CIP2A* in 1880 OvCa patients. Only 4% of patients had alterations in the gene, which were either amplifications or missense mutations of unknown significance. On further investigation, three missense mutations were identified, R530T, E840K, and M6281, from three different patients, were associated with OvCa. Structural analysis of the CIP2A protein indicates that the residues E840 and M6281 are located along the unstructured helical segment, whereas R530 is situated within a critical globular domain (Fig. 2a). The specific position of R530 is particularly noteworthy due to its potential role in dimerisation. A mutation at this site, such as R530T, is predicted to cause structural destabilisation owing to its integral role in maintaining the integrity of the globular domain (Fig. 2b). Consequently, the R530T mutation may directly disrupt dimerisation processes without impacting small molecule binding, given its considerable distance from other identified binding sites, as was described in a ligand binding study of lapatinib with CIP2A^24^ (Fig. 2a). This underscores the mutation’s potential effect on the structural and functional dynamics of CIP2A. In addition, using data from UK Biobank, no significant genetic associations were found between *CIP2A* and OvCa. The associations were primarily with reticulocyte and lymphocyte counts, as well as thyroid function (hypothyroidism; Supplementary Table 1).

### Protein expression of CIP2A in clinical samples

**Fig. 3:**
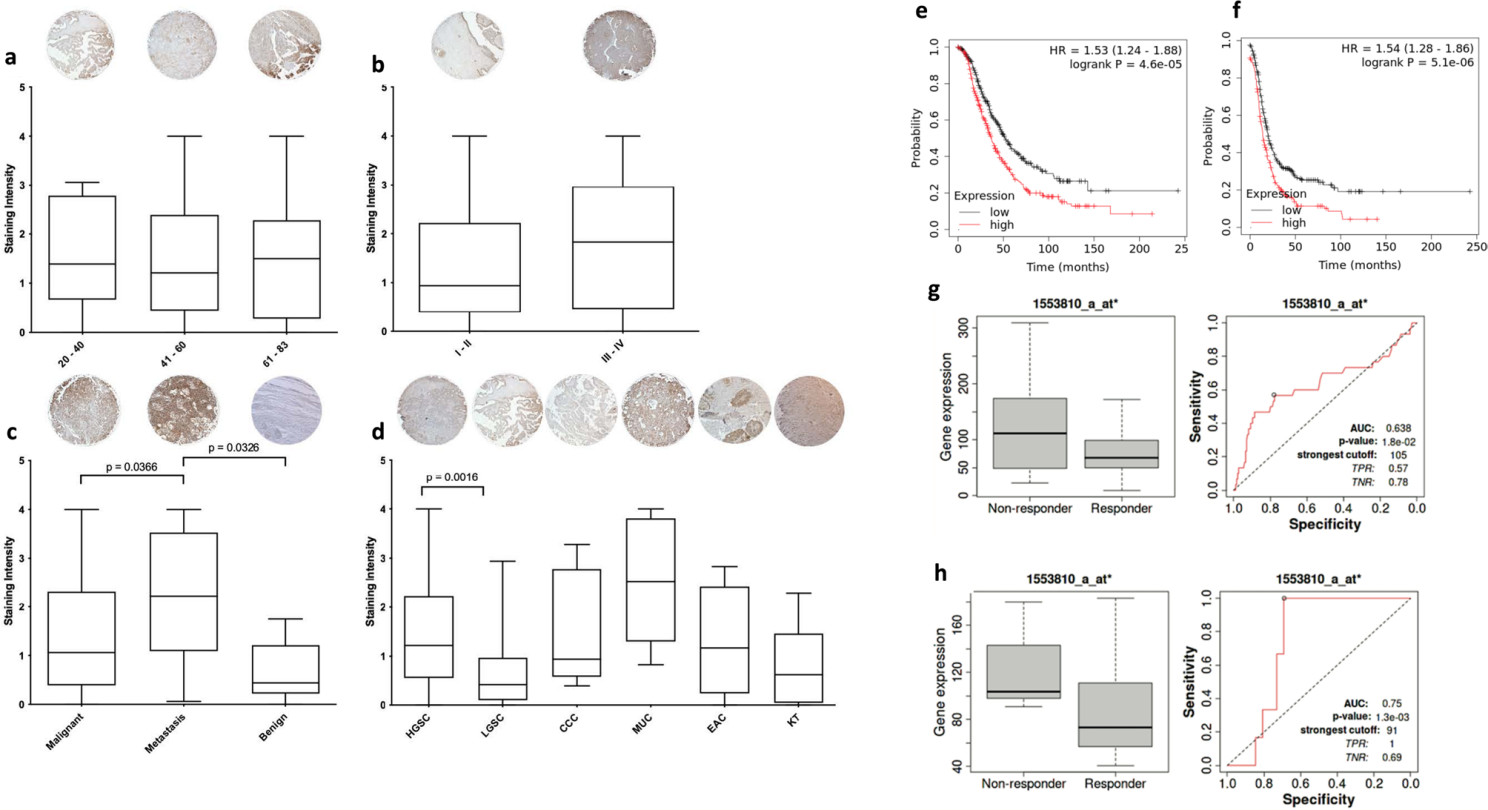
Protein expression of CIP2A depending on the patient’s age (**a**), FIGO stage (**b**), malignant, metastatic and benign status (**c**), and pathological diagnosis: HGS (high-grade serous), LGS (low-grade serous), CCC (clear cell carcinoma), MUC (mucinous), EAC (endometrioid adenocarcinoma), KT (Krukenburg tumour), and LN (lymph node) (**d**). The images of the cores presented within the figure are representative of the group and were captured at 10x magnification. Overexpression of CIP2A is associated with poorer OS (**e**) and PFS (**f**). Six-month relapse-free survival for (**g**) stage III serous ovarian cancer (OvCa) patients who underwent platinum chemotherapy treatment (total *n* = 551, responders *n* = 505, and non-responders *n* = 46) and (**h**) stage IV serous OvCa patients who underwent platinum chemotherapy treatment (total *n* = 88, responders *n* = 79, and non-responders *n* = 9; *p* = 0.018 and *p* = 0.0013, respectively).

We have used a tissue microarray of 131 OvCa patients, to study the protein expression of CIP2A in clinical samples. Our results show that although age and stage (grouped as early vs. late) do not affect expression (Fig. 3a, b); metastatic OvCa patients have significantly higher expression of CIP2A when compared to malignant OvCa samples (*p* = 0.0366) and benign ovarian tumours (*p* = 0.0326; Fig. 3c). When we only compared HGS (*n* = 75) to LGS (*n* = 18), a significant increase of CIP2A in HGS was noted (*p* = 0.0016; Fig. 1d) Proteomic data (UALCAN), corroborate the CIP2A overexpression seen in our clinical cohort (UALCAN, data not shown). We have then investigated, using KM plot^25^the impact of *CIP2A* expression on overall survival (OS) and progression-free survival (PFS) in OvCa patients. Increased expression of *CIP2A* causes a significant decrease in OS (*p* = 4.6e-05, *n* = 655, Fig. 3e) and PFS (*p* = 5.1e-06, *n* = 614, Fig. 3f). These findings are fully in line with data published from Westermarck’s lab^16^.

Following the KM plot results, we decided to determine the possible use of *CIP2A* as a biomarker through plotting a Receiver Operating Characteristics (ROC) curve and corresponding gene expression box-plots using ROC plot^26^. The plots represent 6-month relapse-free survival for both grade III stage III and grade III stage IV serous ovarian cancer (OvCa) patients who underwent platinum chemotherapy treatment. These datasets include data from 551 patients (505 responders and 46 non-responders) for grade III stage III and 88 patients (79 responders and 9 non-responders) for grade III stage IV. CIP2A (Fig. 3g and h) shows an area under curve (AUC) of 0.638 (*p* = 0.018) for grade III stage III and 0.75 (*p* = 0.0013) for grade III stage IV OvCa patients respectively.

### Protein expression of CIP2A is significantly increased in multiple pathways

We have expanded on these observations by employing in silico tools to further investigate protein expression of CIP2A, based on the mutation status of key signalling pathways involved in OvCa. These data are collated by the National Cancer Institute’s Clinical Proteomic Tumor Analysis Consortium (CPTAC), and refer to pathway-level somatic alterations (by small mutation or copy number alteration) in OvCa with combined proteomic, whole-exome, and copy number alterations data. These include RTK (Supplementary Fig. 2a), NRF2 (Supplementary Fig. 2b), WNT (Supplementary Fig. 2c), SWI-SNF Supplementary (Supplementary Fig. 2d), MYC/MYCN (Supplementary Fig. 2e), p53Rb-related (Supplementary Fig. 2f), HIPPO (Supplementary Fig. 2g), mTOR (Supplementary Fig. 2h) and chromatin modifier status (Supplementary Fig. 2i). Protein expression of CIP2A is significantly increased in all tested mutated pathways when compared to normal samples. In addition, p53Rb-related and mTOR pathway mutations demonstrate a significant increase in CIP2A expression when compared to non-mutated pathways with p-values of 0.0297 and 0.0426, respectively.

### Identification of miRNA: mRNA interactions and investigation into their impact

**Fig. 4:**
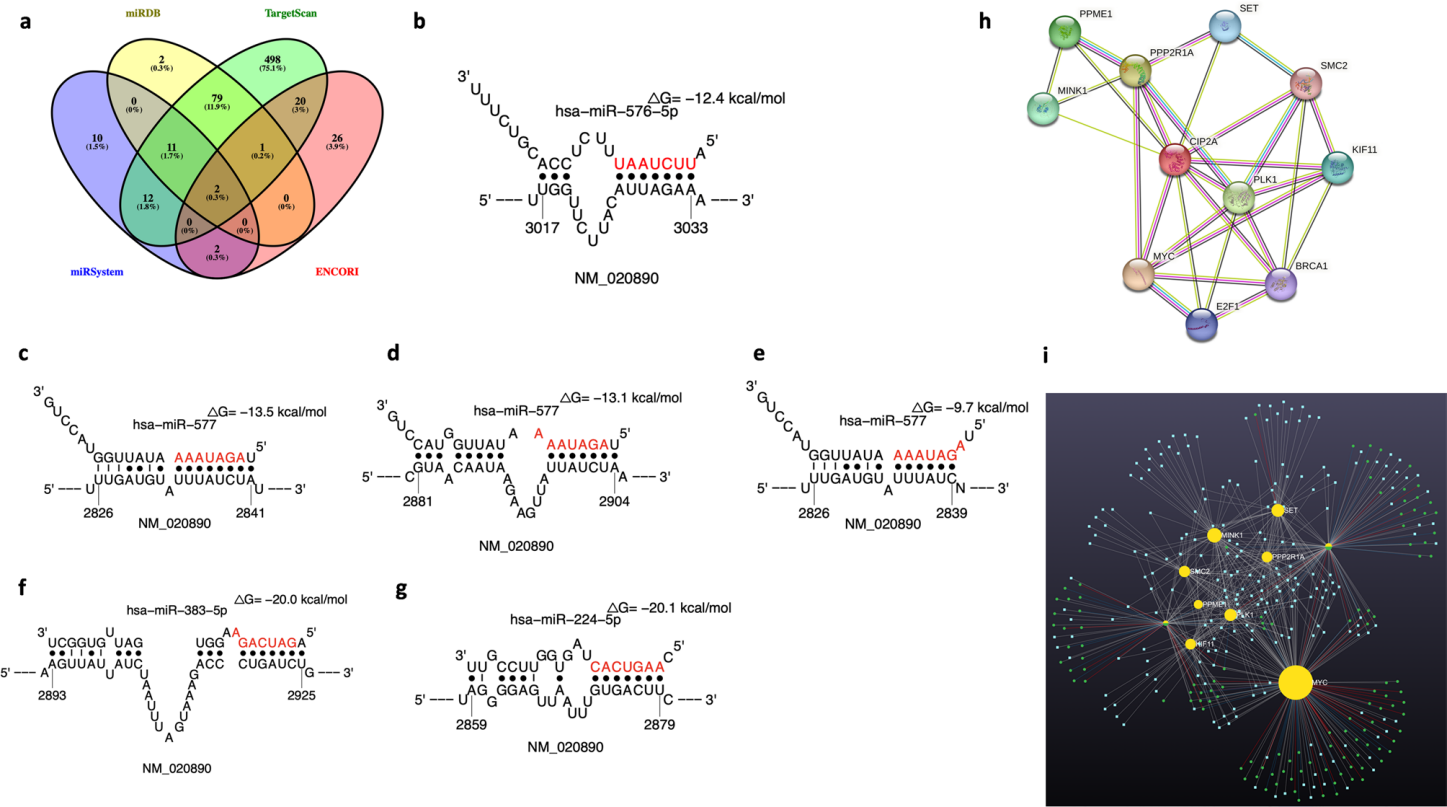
miRNA: *CIP2A* interactions. (**a**): A Venn diagram representing the results from a bioinformatics analysis based on four predictive miRNA-mRNA databases (miRDB, TargetScan, miRSystem, ENCORI), (**b**): Sfold analysis displaying the interaction between miR-576-5p (red) and *CIP2A* (black). Evidence of binding at the 3’UTR at position 3017 − 303 with a ΔG_hybrid_ of -12.4 and an 8mer seed type, (**c-e**): Interactions between miR-577 (red) and *CIP2A* (black). Three binding sites between miR-577 (red) and *CIP2A* (black) were identified via the Sfold analysis at the 3’UTR at positions 2826–2841, 2881–2904, and 2826–2839, with a ΔG_hybrid_ of -13.5, -13.1, and − 9.7 respectively. These also show a seed type of 8mer, 7mer-A1, and offset-6mer respectively, (**f**): Sfold analysis displaying the interaction between miR-383-5p (red) and *CIP2A* (black). One binding site was identified at the 3’UTR at position 2893–2925, with a ΔG_hybrid_ of -20 and a seed type of 6mer, (**g**): Sfold analysis displaying the interaction between miR-224-5p (red) and *CIP2A* (black). One binding site) identified at the 3’UTR at position 2859–2879, with a ΔG_hybrid_ of -20.1 and a seed type of 7mer-m8, (**h**): String analysis depicted ten proteins interacting with CIP2A. These proteins were encoded by the genes: *MYC*,* E2F1*,* BRCA1*,* PLK1*,* MINK1*,* PPME1*,* PPP2R1A*,* SET*,* SMC2*, and *KIF11*, and (**i**): miRNA bundles depicted by miRNet bioinformatics analysis regulating the genes interacting with *CIP2A*. Please note none of the miRNAs regulating/interacting with the ten genes was depicted during miRNA: mRNA interaction analysis performed by miRDB, TargetScan, miRSystem, ENCORI, and Sfold (yellow circles - genes; blue/green circles - miRNAs).

To identify miRNA: mRNA interactions, an investigation of four predictive databases was undertaken (supplementary Fig. 3). These were miRDB, TargetScan, miRSystem, and ENCORI, which identified 95, 623, 37, and 51 interactions respectively (Fig. 4). The results showed that there were two common miRNAs (miR-576-5p and miR-577) across all four databases (0.3%) that were targeting *KIAA1524*.

Following on, we evaluated their strength of binding to *CIP2A* through Sfold binding analysis. Primarily, the interaction between miR-576-5p and *CIP2A* was investigated. This analysis showed that there is only one binding site between miR-576-5p (red) and *CIP2A* (black) at the 3’UTR (untranslated region) at position 3017–3033 with a ΔG_hybrid_ of -12.4 (Fig. 4b, Table 1). As well as this, the seed type identified was 8mer, signifying that the miRNA potentially binds to *CIP2A* with full complementarity and could lead to its complete degradation. Sfold analysis was also used to examine the interactions between miR-577 and *CIP2A*. The results showed three binding sites between miR-577 and *CIP2A*. The most substantial primary interaction, at the 3’UTR at position 2826–2841, with a ΔG_hybrid_ of -13.5, and a seed type 8mer. The second interaction is at the 3’ UTR at position 2881–2904, with a ΔG_hybrid_ of -13.1, and a seed type 7mer-A1. The third interaction is at the 3’UTR at position 2826–2839, with a ΔG_hybrid_ of -9.7, and a seed the offset-6mer (Fig. 4c-e; Table 1). The presence of multiple binding sites between miR-577 and *CIP2A* suggested a greater modulation of gene expression by this miRNA.

**Table 1:**
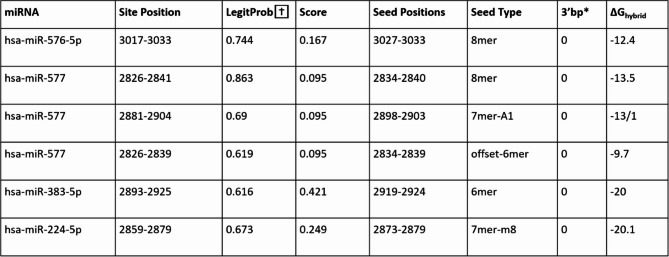
Sfold binding analysis between miR-576-5p, miR577, mR-383-5p, mir-224-5p and *CIP2A*.

TargetScan identified two additional miRNAs targeting *CIP2A* with great affinity: miR-383-5p and miR-224-5p (Table 2). These were further investigated with Sfold. The analysis highlighted one binding site between miR-383-5p (red) and *CIP2A* (black) at the 3’UTR at position 2893–2925, with a ΔG_hybrid_ of -20 and a seed type of 6mer. The study of the interaction between miR-224-5p (red) and *CIP2A (black)* showed that the binding occurs at the 3’ UTR at position 2859–2879, with a ΔG_hybrid_ of -20.1 and a seed type of 7mer-m8 (Fig. 1f-g). The protein interactome analysis of CIP2A demonstrated its interaction with 10 proteins, encoded by the following genes: *MYC*,* E2F1*, *BRCA1*,* PLK1*,* MINK1*,* PPME1*,* PPP2R1A*,* SET*,* SMC2*,* and KIF11* (Fig. 4h-I, and Supplementary Table 2).

**Table 2:**
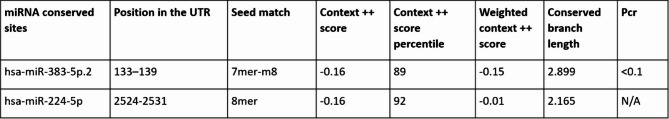
TargetScan conserved mirna: mRNA binding interaction analysis depicted two MiRNAs targeting CIP2A with great affinity: miR-383-5p and miR-224-5p, respectively.

Using the database of Differentially Expressed MiRNAs in human Cancers (dbDEMC)^27^ and making use of publicly available patient-derived datasets, we demonstrate that miR-576-5p and miR-383-5p are downregulated in OvCa (logFC: -2.68 and − 7.36 respectively; datasets GSE65819 and GSE119055). miR-577 demonstrated a weak increase (logFC: 0.68; dataset GSE106817) and miR-224-5p appears to be upregulated in OvCa (logFC: 5.30; dataset GSE83693).

### Inhibition of CIP2A using TD-19

**Fig. 5:**
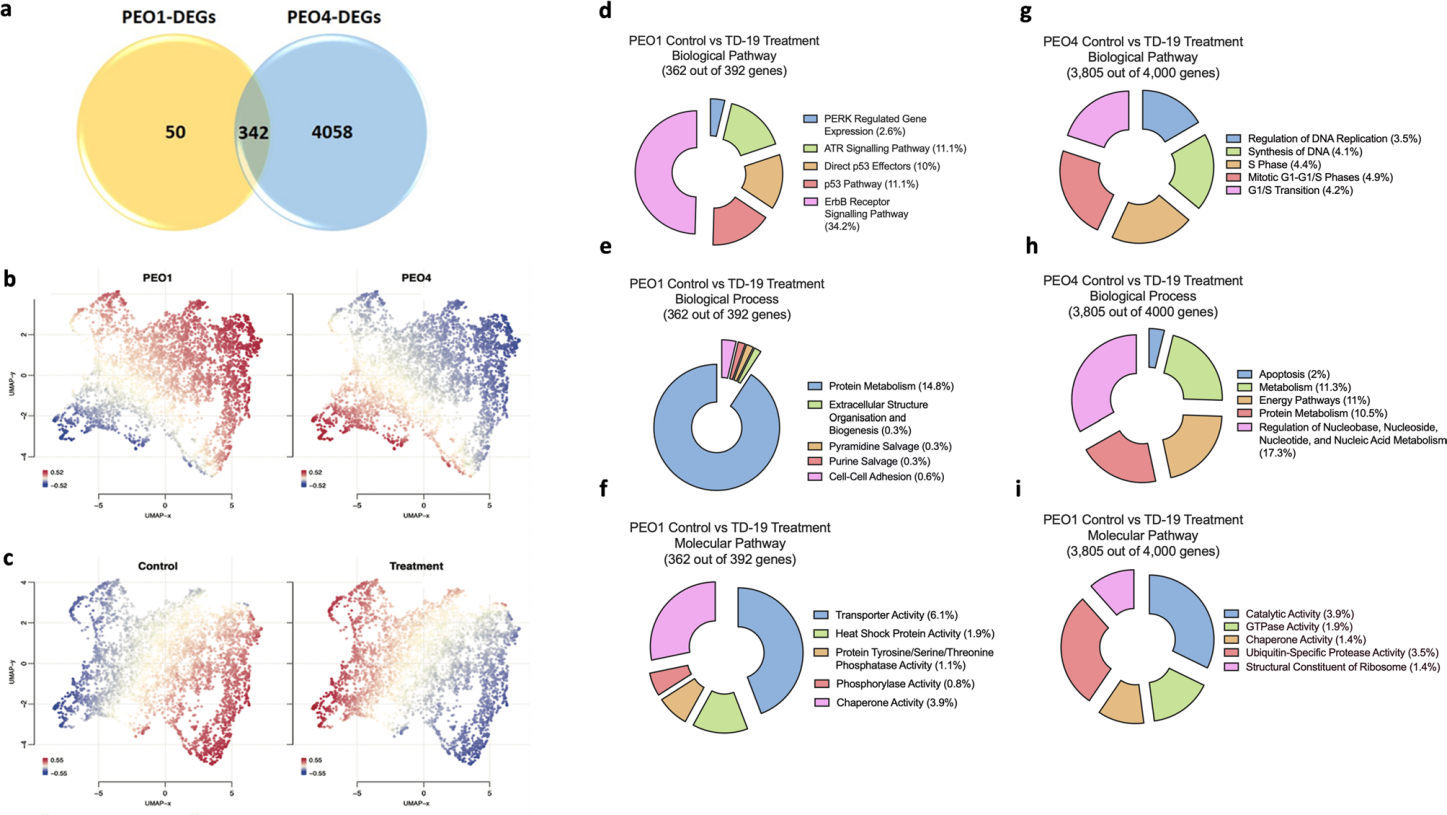
Differentially expressed genes and enriched biological pathways, processes and molecular functions associated with TD-19 treatment in PEO1 and PEO4 cells. **a**) A Venn diagram displaying the differentially expressed genes (DEGs) for TD-19 treated PEO1 and PEO4 samples when compared to their respective controls, (**b-c**) Geneset signature uniform manifold approximation and projection displaying up and down-regulated genes in different samples. UMAPs display genes clustered by relative log-expression which were up (red) or down (blue) regulated in PEO1 and PEO4 (b), no supplement (control) and treated samples (c). Images produced using Omics Playground. Top 5 enriched (**d**) and (**g**) Biological Pathways, (**e**) and (**h**) Biological Processes, and (**f**) and (**g**) Molecular Pathways for PEO1 and PEO4 cell lines respectively, treated with TD-19.

We used the TD-19, an Erlotinib derivative marketed as a transcriptional CIP2A inhibitor, to gain a better insight into the role of CIP2A in an in vitro BRCA2 model (PEO1) compared to BRCA2 wild-type (PEO4). RNA sequencing revealed that TD-19 induced cell-specific differential expression for 50 genes in PEO1 cells, and 4058 in PEO4, as well as pointing at 342 DEGs in both systems (Fig. 5a, Supplementary Tables 3 and 4). A t-distributed stochastic embedding (t-SNE) plot based on the DEG data demonstrated a robust discrimination, both between PEO1 and PEO4 samples and between TD-19 treated and control samples (Supplementary Fig. 4a). TD-19 treatment (5µM for 24 h), downregulated CIP2A primarily in the BRCA2 wild type (wt) cell line, PEO4 (Supplementary Fig. 4b-d). A Uniform Manifold Approximation and Projection (UMAP) plot was created to display the up- (red) and down-regulated (blue) genes for PEO1 and PEO4 samples (Fig. 5b) and TD-19 5µM treated and control samples (Fig. 5c). The results show a complete contrast between the two cell lines as well as between treated (both cell lines combined) and control samples (i.e. untreated).

### Gene enrichment analysis

Funrich was used to determine the top five enriched biological pathways (Fig. 5d), biological processes (Fig. 5e), and molecular pathways (Fig. 5f) in PEO1 cells treated with TD-19. The results show involvement in p53 pathway, protein metabolism, and transporter activity. Interestingly, the rest of the biological pathways are related to kinase activity, two of which are known to be important in cancer (ErbB and ERK). To further corroborate this under molecular pathways protein tyrosine/serine/threonine kinases were also highlighted. For biological processes, cell-cell adhesion was one of the five which is important and could indicate a relationship with EMT and thus metastasis. Similar analyses were performed for the PEO4-related DEGs, identifying the top five enriched biological pathways (Fig. 5g), biological processes (Fig. 5h), and molecular pathways (Fig. 5i). Of note, all of the biological pathway data show involvement in DNA replication, and the cell cycle. Regulation of nucleobase, nucleoside, nucleotide, and nucleic acid metabolism are also part of the top five biological processes. For molecular pathways, the top two were catalytic and GTPase activity. All p-values for the top five enriched biological pathways, biological processes, and molecular pathways (depicted in Fig. 5) were significant (Supplementary Fig. 5a-b).

### Heat map of the top 150 DEGs identifies four different clusters

**Fig. 6:**
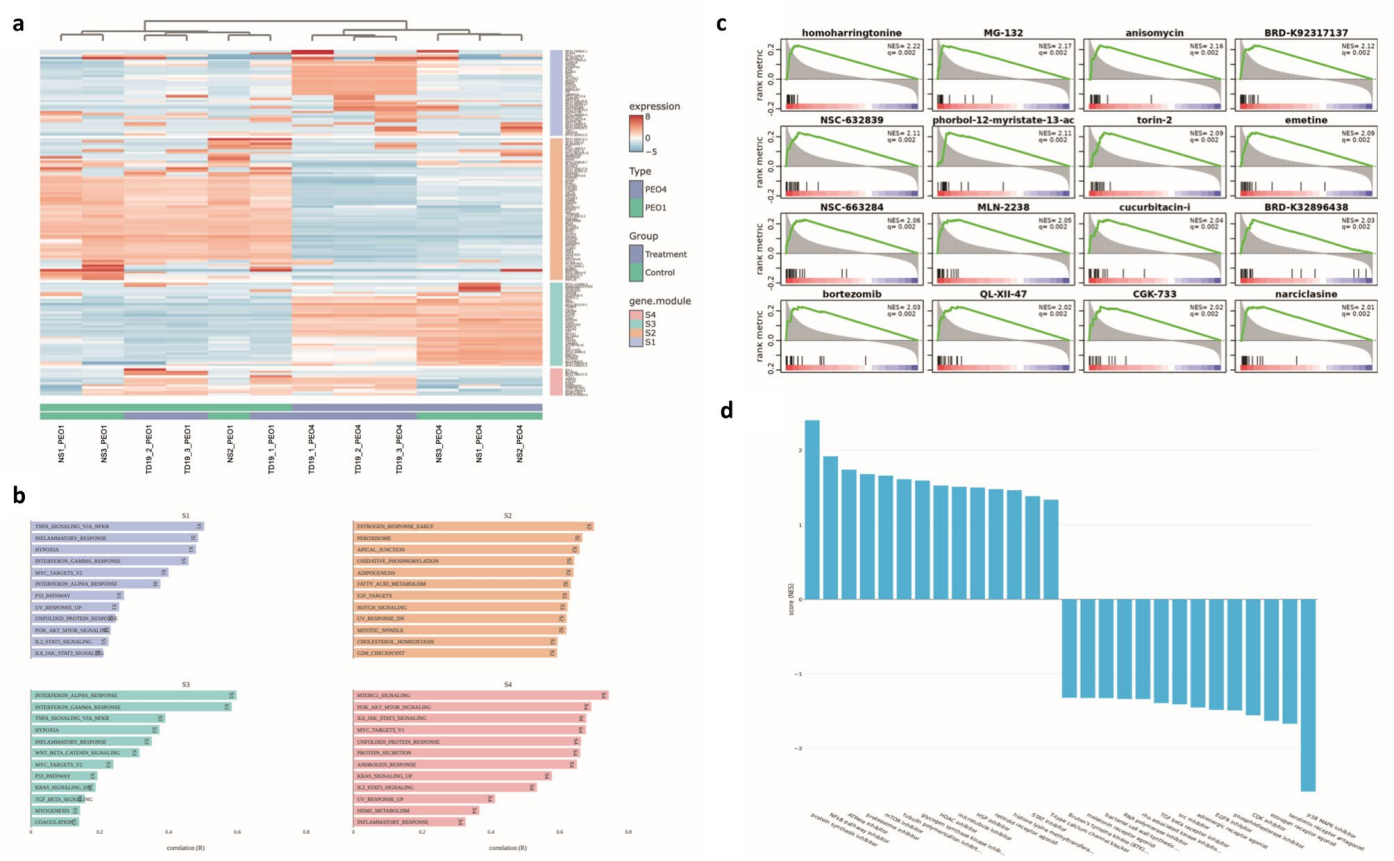
*Clustered heatmap and functional annotation.* **a**) Functional heat map of 150 genes identified through the highest standard deviation across all samples (PEO1 vs. PEO4 and treatment vs. control). The hierarchical clustering was performed at the gene level and using the relative expression scale. In this heatmap, red signifies over- expression and blue under-expression. There are four clusters S1 (blue), S2 (orange), S3 (green), and S4 (red). **b**) The functional annotation terms for each of the four clusters identified in (**a**). These were generated through the use of Omics Playground which correlates each gene set to over 42 reference databases such as KEGG and Gene Ontology. *Drug connectivity and mechanisms of action.* **c**) The correlation between the TD-19 treatment data and known drug profiles from the L1000 database. Here, each of the drugs from the databases shows a similar gene dysregulation pattern observed with TD-19 treatment. Each of the bars represents a gene; red signified up- regulation and blue down. **d**) A plot displaying the most correlated mechanisms of action (MOA) across the enriched drug profiles identified from the L1000 activities database compared to TD-19. The Gene Set Enrichment Analysis (GSEA) normalised enrichment score is on the y-axis and the MOA is on the x-axis.

Figure 6a depicts the functional heat map of the top 150 genes identified based on differential expression pattern with the highest standard deviation across all samples ([PEO1 vs. PEO4 and treatment] vs. control). The hierarchical clustering was performed at the gene level and showcased four clusters S1-S4 (Fig. 6b). Many of these functional annotations are related to cancerous pathways such as the TNF-α signalling via NF-kB, MYC targets, and p53 pathway in S1, Oestrogen response early, E2F targets, and G2M checkpoint in S2, Interferon alpha and gamma response in S3 (TNF-α signalling via NF-kB also appears in this cluster), and mTOR signalling, PI3K-Akt-mTOR signalling and MYC targets in S4.

### TD-19 treatment acts similarly to other drug treatments

Omics Playground allows for the determination of correlation between dysregulated genes as a result of TD-19 treatment and as a result of other known drug profiles from the L1000 database^28^. In essence, this analysis identifies which drugs can give a similar genetic signature to TD-19. Figure 6c shows the results for this with 16 top drug profiles shown, with the closest profile to TD-19 being Homoharringtonine, an anti-cancer drug already in clinical use. Some other compounds of interest were the mTOR inhibitor (Torin-2) and another clinically used anti-cancer drug Bortezomib . Following on from this, the most correlated mechanisms of action (MOA) across the enriched drug profiles identified from the L1000 activities database compared to TD-19 were determined and are shown in Fig. 6d. We selected the positively correlated MOA of interest as: NFkB pathway inhibitor, mTOR inhibitor, and protein synthesis inhibitor, and the negative are RNA polymerase inhibitor, TGFβ receptor inhibitor, EGFR inhibitor, oestrogen receptor agonist, and p38 MAPK inhibitor.

We have also interrogated the correlation of expression patterns between *KIAA1524* and other genes, using Omics Playground^29^and the results are shown in Supplementary Fig. 6. Both conditions tested (i.e. PEO1 and PEO4), show strong positive correlations with *CIP2A* and *EPCAM*,* POLB*,* MFSD8*,* and OSTF1*.

### Effects of TD-19 on cell proliferation

In order to investigate the effects of TD-19 on cell proliferation, we have used the ovarian carcinoma cell line SKOV-3 as a preclinical model. We have decided to use this cell line as it is one of the most frequently used OvCa in vitro models (both 2D and 3D)^30^. Moreover, when we performed RT-qPCR, we demonstrated that SKOV-3 cells express higher amounts of *CIP2A* when compared to PEO1 (*p* < 0.0001) or PEO4 (*p* = 0.0075) cell lines (data not shown); thus, making it an ideal target for TD-19. A wound healing assay was initially used to explore the migratory and proliferative capacity of TD-19 treated SKOV-3 cells over 72 h (5µM). The gap closure was delayed in the treated cells when compared to control cells (no treatment; Supplementary Fig. 7a-b). This was corroborated by a cell viability assay, demonstrating that SKOV-3 cells treated with TD-19 demonstrated a significant decrease (*p* < 0.001) in numbers of live cells over 24–72 h (Supplementary Fig. 7c). Furthermore, there was an increase in the apoptotic population (early apoptosis; annexin V+/PI−, late apoptosis; annexin V+/PI+) in treated cells over 24–72 h (Supplementary Fig. 7d).

## Discussion

In this study we provide a comprehensive overview of the role of CIP2A in OvCa. Our data corroborate previous studies that demonstrated overexpression in 39–90% of malignancies, including OvCa^11,15^. *CIP2A* upregulation in OvCa was independent of stage or age, but significantly overexpressed in OvCa patients that have TP53 mutations. Indeed, previous studies have shown that *CIP2A* expression is stimulated by TP53 mutation in head and neck cancer^31^and its inactivation (via the P21-E2F1 axis) modulates *CIP2A* expression^9,19^. We also show an increased expression of CIP2A in patients who have mutations in their mTOR-pathways when compared to when compared to non-mutated pathways patients and healthy controls. CIP2A is known to affect mTOR complex 1 (mTORC1) signalling which not only inhibits autophagy but also increases cell growth and proliferation^11^. Puustinen et al., have also linked CIP2A with mTOR activity, since it promotes mTORC1-mediated cell proliferation and inhibition of autophagy^32^.

Using tissue microarrays, we demonstrate aberrant expression of CIP2A in OvCa patients, independent of age or stage (early vs. late). Emerging spatial transcriptomics data corroborates similar expression. We also provide evidence for overexpression of CIP2A in metastatic patients when compared to primary tumour samples indicating its potential involvement as an oncoprotein in the progression of the disease. This finding agrees with the study by Böckelman et al.^33^. , where they demonstrate that increased CIP2A expression is associated with more aggressive OvCa and worsen survival. The KM plots presented here corroborate this further, since increased expression of *CIP2A* in OvCa patients, both serous and endometrial, was associated with significantly poorer OS and PFS. Further verification comes from investigations into many other solid tumours, which indicate that increased expression of CIP2A is associated with poorer outcomes^8^. We have also shown that mucinous OvCa has a high CIP2A expression. Of note, there is a high percentage of patients with TP53 mutations, suggestive of potential involvement in the upregulation of CIP2A in mucinous OvCa^34^.

Little is known about the impact of CIP2A mutations in health and disease. For example, the activating CIP2A p.D269V mutation, is a novel genetic cause for this malignancy, as it drives increased expression of PP2A, mTOR, and c-Myc^35^. Loss of function variant in CIP2A (c.1510 C > T, p.L504F), has also been identified as a cause for early embryonic arrest and fragmentation, from a consanguineous family^36^. UK Biobank analysis did not reveal any associations of polymorphisms with OvCa. It appears therefore, that the protein is so widely over expressed in cancer by so many oncogenic pathways that there is no need for mutations, and overexpression is enough as an oncogenic driver. However, in this study we identify that the R530T mutation presented in an OvCa patient, can potentially cause structural changes affecting the dimerisation of this molecule. To further elucidate the impact of the R530T mutation and other mutations on the CIP2A structure and function, several advanced computational studies could be undertaken in future studies. Future molecular dynamics (MD) simulations could be employed to observe the dynamic behaviour of both the wild-type and mutant CIP2A proteins, providing insights into conformational changes, stability, and interactions over time. This could enhance our understanding of CIP2A’s structural and functional dynamics and guide experimental validation studies. Despite the lack of direct association between *CIP2A* and OvCa, based on UK Biobank data, it is worth mentioning that other associations provide a novel insight into the role of this gene. For example, a genome-wide association study (GWAS) for certain endocrine indices, found a causal relationship between OvCa and thyroid function^37^whereas late stage OvCa patients were found to have significantly lower reticulocyte counts when compared to healthy controls^38^.

Our miRNA: mRNA in silico analysis showed that *CIP2A* was a direct target for miR-576-5p and miR-577. A recent study observed that a circular RNA called hcR1445 reduced cellular invasion, proliferation, and migration by inhibiting miR-576-5p, suggesting the overexpression of miR-576-5p in OvCa and its action as an oncomiR^39^. The role miR-576-5p has also been related to the upregulation of *SFRP1* expression, which in turn activates WNT/β-catenin signalling, promoting OvCa progression^39^. Our Sfold analysis depicted one binding site between miR-576-5p and CIP2A, hinting at a potential regulatory function for the miRNA. In miRNA-mediated upregulation, microRNA ribonucleoproteins (miRNPs) can act in trans to enhance the expression of their target mRNAs. This upregulation can occur through the direct action of miRNPs on the mRNA or by indirectly relieving the mRNA from repression by inhibiting the activity of repressive miRNPs^40^. Thus, we could hypothesise that miR-576-5p has protective mechanisms upon *CIP2A*, preventing post-translational repression or degradation. Nevertheless, the expression of miR-576-5p in OvCa has not been deeply studied and further research is necessitated to identify its exact molecular mechanisms and mode of action.

On the other hand, our four-database miRNA: mRNA interactome analysis showed that *CIP2A* was also targeted by miR-577. Previous research has demonstrated that the expression of miR-577 in OvCa cells was significantly downregulated^41^. Additionally, miR-577 was identified as a direct downstream target for a long intergenic non-coding, *LINC01094*. Inhibition of miR-577 led to the expression of *LINC01094* and enhanced its proliferative effects upon OvCa cells, suggesting the tumour-suppressive properties of miR-577 in OvCa^41^. Our analysis also depicted three binding sites between miR-577 and *CIP2A*, suggesting a robust and efficient regulation of the mRNA, ensuring precise control over gene expression by promoting mRNA degradation and inhibiting tumour growth.

Furthermore, we identified that miR-383 was also targeting *CIP2A*. This interaction has been previously demonstrated in glioma cells, where miR-383 inhibited the expression of *CIP2A* and seized the proliferation and invasion of the cancer cells^42^. The significant downregulation of miR-383 in OvCa has also been observed^43^. Even though the interaction between miR-383 and *CIP2A* has not been previously reported in OvCa, we could suggest that miR-383 could exert its tumour suppressive properties via targeting the genes, consequently leading to tumour growth inhibition. In contrast, the upregulated expression of miR-224-5p in OvCa has been observed^44^. Nevertheless, its interactions with *CIP2A* have not been elucidated before. Based on the data generated from dbDEMC, the observed downregulation of miR-576-5p and miR-383-5p supports our hypothesis that loss of these two miRNAs might relieve suppression on *CIP2A*, leading to its upregulation. Considering that these findings are derived from clinical samples, this data strengthens the idea of a regulatory axis between these two miRNAs and *CIP2A* in a biologically relevant context.

Inhibition of CIP2A with TD-19 in BRCA2 mutant and wild-type preclinical OvCa models, provided a novel insight into transcriptomic changes. We have chosen these in vitro models, since it has been shown that in cells that are BRCA deficient, the CIP2A forms a complex with TOPBP1 that subsequently prevents lethal mis-segregation of chromosomes (lacking centromeres), due to impaired DNA synthesis^45^. TD-19, an Erlotinib derivative, is marketed as a CIP2A inhibitor. TD-19 differs from Erlotinib as 4-phenoxyaniline is added at position 2 of quinazoline. This prevents TD-19 from binding via hydrogen bond to EGFR ATP-binding site as Erlotinib does^46^. TD-19 has been shown to be effective at inducing apoptosis in non-small cell lung cancer (NSCLC) cell lines at a concentration of 5–10µM^46^and in mouse testicular cells, CIP2A was significantly down-regulated after treatment with TD-19^47^. Similarly, we also demonstrate a decrease of cell proliferation in vitro, following TD-19 treatment.

Functional enrichment in Gene Ontology terms, was performed in relation to the top 5 biological pathways, biological processes, and molecular pathways for both cell lines, treated with TD-19. In terms of biological pathways (PEO1, BRCA2 mutant), p53 pathway was highly enriched. Although there is some evidence of an interaction between CIP2A and p53, the whole mechanism is not fully elucidated yet. This is not a surprise, given the links between TP53 and CIP2A.*TP53* is mutated in over 96% of cases in high-grade serous OvCa^48^. We also provide evidence for potential interactions with ErbB receptor and ATR signalling pathway. Of note, overexpression or activation of ErbB receptors, has been linked with poor prognosis and metastatic events^49^. ATR signalling tends to get activated when the genome responds to single-stranded DNA gaps, and in OvCa, interruption of ATR signalling further sensitised BRCA1/2-deficient OvCa cells to PARP inhibition^50^. In terms of biological processes, the most enriched pathway was that of protein metabolism. Indeed, in OvCa, metabolic alterations are key in order for cancer cells to sustain uncontrolled proliferation, and rebuild biomasses including proteins^51^. In the BRCA2 wild-type PEO4 cells, the top five enriched biological pathways were all shown to be involved in the regulation of the cell cycle. CIP2A has been shown to be involved in promoting cell cycle progression in triple-negative breast cancer (TNBC) cells, whereas its inhibition, leads to cell cycle arrest in hepatocellular carcinoma cells^52^.As mentioned previously CIP2A is also involved in the cell cycle progression through interactions with for example Plk1 or E2F1^9,53^. An analogous enrichment pattern to PEO1 cells was also noted, in terms of biological processes, with metabolism and energy pathways being the predominant ones. Heat map of the top 150 DEGs, identified similar clusters to the enriched pathways, including PI3K-Akt-mTOR signalling and MYC targets. Finally, a strong correlation was identified with CIP2A and EpCAM, a transmembrane glycoprotein that is over-expressed in many cancers including ovarian, breast, and lung^54^. It is a cell adhesion molecule, playing an important role in EMT and metastasis^55^. This provides a further link to the biological processes data for PEO1 in terms of cell- cell adhesion.

The drug screening provided a wide repertoire of compounds which act similarly to TD-19 displaying a comparable molecular signature. These results show that many small molecule inhibitors of proteases (MG-132, MLN- 2238) and CIP2A interacting proteins such as mTOR inhibitors (Torin-2) were identified, which have been studied for their possible use for cancer treatment, yielding positive results in vivo^56^. Perhaps more interestingly, two of the identified drugs are clinically used drugs. For example, Homoharringtonine is used for chronic myeloid leukaemia and has been shown to suppress growth and induce apoptosis in TNBC^57^NSCLC^58^and hepatocellular carcinoma^59^. Bortezomib is also used for treating multiple myeloma patients and works as a proteasome inhibitor^60^. Collectively these data point towards potential drug repurposing. Future studies should concentrate on elucidating whether any of the compounds described in this study can exert an inhibitory effect on CIP2A signalling. Moreover, a recent study by Avelar et al., has shown that using a small-molecule activator of PP2A termed SMAP-061, appears to potentiates DNA damage-induced cell death in patient-derived HGSC cells and xenograft models alone or in combination with PARP inhibitors^61^.

## Conclusion

This study provided a deeper insight into the role of CIP2A in ovarian cancer making use of a wide repertoire of approaches. We provide further evidence of the regulation of signalling mechanisms using a CIP2A inhibitor, and identified compounds that can exert similar effects. We also acknowledge that out study has certain limitations. For example, given that HGSC is characterised by p53 mutations and accounts for the majority of high-grade ovarian tumours, it would be of interest to identify which cohorts of patients carry a p53 mutation and subsequently have a higher expression of CIP2A. Moreover, we have used a small cohort of patients for this study, and have not validated the impact of TD-19 on certain signalling pathways in vitro. Future studies should concentrate on increasing the TD-19 testing on different preclinical models of OvCa (e.g. endometrioid, early stage, etc.), and then move to in vivo studies, such as using models of subcutaneous tumorigenesis in nude mice. Given the increasing clinical utility of liquid biopsies, it will also be useful to study the expression of CIP2A in circulating tumour cells, and its gene expression in total RNA (from whole blood) as well as circulating-free RNA.

## Methodology

### In silico CIP2A (KIAA1524) analyses

The GTEx Portal^10^ was used to identify the variation in *CIP2A* expression in normal breast and gynaecological tissues. The online tool OncoDB^17^ was used to investigate *CIP2A* expression in OvCa in comparison to normal healthy tissues. As well as this, the online tool UALCAN^20^ was used to allow for analysis of *KIAA1524* gene and CIP2A protein expression in cancers. CbioPortal^23^ was used to determine common mutations on the CIP2A gene and determine the location of these mutations. Alphafold2^62^ was used to predict the protein structure of CIP2A and to determine per-residue confidence scores (PLDDT) of specific locations on the protein. The protein structures for CIP2A were processed and images generated using the VMD (Visual Molecular Dynamics) software^63^.

The tool KM plot^64^ was used to determine the impact of the expression of genes of interest on overall survival (OS) and progression-free survival (PFS) in OvCa. The tool ROC Plotter^26^ was used to generate ROC plots to determine the predictive value of CIP2A as a biomarker in OvCa. For the genotyping study, UK Biobank (a large population database of around half a million individuals) was used as previously described^65,66^.

### In silico miRNA analysis

The following analysis was performed as previously described by Mustafov et al., 2024^67^. In brief the databases, miRDB^68^TargetScan^69^miRSystem^70^and ENCORI^71^ were employed to identify functional miRNA: mRNA target interactions (MTIs). Following this, the results from the four databases were plotted in a Venn diagram using Venny2.1.0^72^ to identify the more efficacious MTIs and eliminate the chance of false positive results. The database miRbase^73^ was used to obtain miRNA sequences and annotations. Following this, the database Sfold^74^ was utilised to determine the probable binding sites of the identified miRNAs to their gene target. The database STRING^75^ was used to identify the top co-expressed genes with *CIP2A* and these were used to plot a network ofMTIs with *CIP2A* and its co-expressed genes using miRNET2.0^76^.

### Cell culture

PEO1 and PEO4 cell lines were used to model OvCa in vitro. These two cell lines were specifically chosen as they originate from the peritoneal ascites of the same patient who presented with poorly differentiated serous adenocarcinoma. Primarily, the patient had a BRCA2 mutation ((5193 C > G (Y1655X) (PEO1) and went on to relapse and present with a novel mutation which caused restoration of BRCA2 (5193 C > T (Y1655Y)) (PEO4). Both cell lines were cultured in Roswell Park Memorial Institute (RPMI) medium 1640 and GlutaMAX (61870-010, Gibco) supplemented with 10% foetal bovine serum (FBS) (10270-106, Gibco) and 1% penicillin-streptomycin (p/s) (15070-063, Gibco). Cells were incubated at 37 °C with 5% Carbon Dioxide (CO2) and passaged around two to three times a week when approaching 80% confluence using TrypLE™ Express Enzyme (12604013, Gibco). For functional experiments, the SKOV-3 cell line (ECAAC 91091004) was grown in Dulbecco’s modified Eagle’s medium (DMEM), as previously described^77^.

### TD-19 treatment

The inhibitor TD-19 (5329120001, Sigma-Aldrich) was dissolved in DMSO, aliquoted and stored at -20 °C. PEO1 and PEO4 cells were seeded at 80% confluence and left until a confluent monolayer formed (around 1 day) with 2mL of media. Once confluent, the media was replaced with 2mL of fresh pre-warmed media and treated with 5µM of TD-19. The no-supplement cells were treated with the same amount of DMSO. The 6-well plates were then placed in the incubator at 37 °C with 5% CO^2^. After 24 h of treatment, protein extraction for western blots and RNA extraction were performed.

### RNA extraction, sequencing, and analysis

Following TD-19 treatment, RNA extraction on PEO1 and PEO4 cells was performed using the RNeasy^®^ Mini Kit (74104, Qiagen) following the manufacturer’s instructions. The isolated RNA was sent on dry ice for sequencing by Novogene, Cambridge, for pair-end sequencing. The Tuxedo Suite software package was used to analyse the RNA-seq results. Pair-end reads were mapped to the human reference genome (GRCh38) using Tophat2 (v.2.1.1) and Bowtie2 (v.2.2.6). Following this, Samtools (v.0.1.19) was used to merge the replicates and filter the aligned reads to a quality threshold of < 30. To assemble and quantify the transcript, GENCODE annotation v26, Cufflinks (v.2.2.1), and Cuffdiff (v.2.2.1) were used to determine the differentially expressed genes (DEGs) of the treated samples compared to the control samples. The online tool FunRich^78^ was used to analyse the involvement of the DEGs as a result of TD-19 treatment in biological processes, molecular functions, and biological pathways. As well as this, the online tool^29^ was used to input the RNA sequencing data to perform various analyses and generate graphs.

### Western blotting

The Western blotting was performed as previously described^77^. In brief, protein lysate extraction was performed using pre-boiled Laemmli buffer (S3401-1VL, Sigma), followed by boiling the samples for 10 min at 100 °C. The gel was cast, samples and ladder loaded, and the electrophoresis was performed at 80 mA constant and 300 V for 50 min. Following this, the wet transfer was performed at 400 mA and 300 V for 90 min. The blocking stage was then performed for 1 h at RT. After this, the primary antibodies were prepared, CIP2A (1:500, Ab84547, Abcam) and GAPDH (1:1000, 2118 S, Cell Signalling) diluted in 5% BSA/TBS-Tween 20 overnight at 4 °C. The following day, the membrane was washed in TBS-Tween 20 three times. The secondary HRP antibody goat anti-rabbit (1:2000, ab205718, Abcam) was diluted in 1:2000 in 5% BSA/TBS-Tween 20 for 1 h on the rocker at RT followed by three washes in TBS-Tween 20. To visualise the bands, the chemiluminescent substrate was made and added to the membrane for two minutes in a dark room. After this, the membrane was transferred to the cassette with an Amersham™ Hyperfilm™ ECL™ (28-9068-36, Cytiva) photo paper placed onto it. The cassette was then closed for one minute then the film placed in the AGFA Curix 60 film developer. The blots were then analysed using densitometric analysis on the software ImageJ Statistical analysis was conducted using a t-test, where *p < 0.05.

### Immunohistochemistry (IHC)

IHC was performed as previously in Filipe et al., 2021^79^. In brief, commercially available, paraffin-embedded tissue microarray slides containing OvCa patient samples and normal adjacent ovarian tissue (NAT) samples were purchased (BC11115d and OV991, US Biomax Inc., Derwood, MD 20855, USA) (Supplementary Tables 5 and 6 respectively). These tissue samples were collected with full consent from the patients, in compliance with Health Insurance Portability and Accountability Act (HIPAA) approved protocols to ensure ethical standards. Use of commercially-available TMAs falls under the HTA licence of Brunel University London (#12543). The slides underwent de-paraffinisation through a series of washes, primarily in Histo-Clear (12358637, National Diagnostics, Loughborough, LE11 5RG, UK), 50:50 Histo-Clear and ethanol, then a serial dilution of ethanol. The antigen retrieval step was performed in heated sodium citrate (BP1325-1, Fisher Bioreagents, Loughborough, LE11 5RG, UK) for 10 min, followed by a series of washes in distilled water and PBS 0.025% Triton-X. The slides were then incubated in 3% hydrogen peroxide for 10 min, followed by three washes in PBS 0.025% Triton-X. For blocking, the slides were placed in a humidity chamber with 5% BSA in PBS for 1 h at RT. Following this, the CIP2A primary antibody (1:100, ab84547, Abcam) in 5% BSA was placed on the slides, in the humidity chamber and incubated overnight at 4 °C. The following day, the slides underwent three washes in PBS 0.025% Triton-X. Then an anti-rabbit secondary antibody (HRP008DAB-RB, Zytomed Systems, Oxfordshire, OX25 5HD, UK) was placed on the slides and incubated for 1 h at RT in a humidity chamber. The slides then underwent three washes in PBS 0.025% Triton-X. Streptavidin-HRP conjugate was added to the slides and incubated for 50 min at RT in the humidity chamber. Following this, the slides underwent washes in PBS 0.025% Triton-X. Then, the DAB solution was prepared and dispensed onto the slides for 10 min. This was followed by a counterstain with Harris’ Haematoxylin. The slides then underwent dehydration through a series of washes in an increasing concentration of ethanol followed by 50:50 Histo-Clear and ethanol then Histo-Clear. The degree of staining of each stained core was scored from 0 to 4 as follows: 0 if < 10% stained, 1 if 10 to 25% stained, 2 if 25 to 30% stained, 3 if 50 to 75% stained and 4 if ≥ 75%. The scoring was repeated three times on three different days and performed by three lab members.

### Cell proliferation assays

A wound healing assay was performed as described by Sotiriadis et al.^80^. Immunofluorescence, cell viability and Annexin V/PI assays were performed following the protocols described by Saravi et al.^81^.

## Electronic supplementary material

Below is the link to the electronic supplementary material.

Supplementary Material 1

Supplementary Material 2

Supplementary Material 3

Supplementary Material 4

Supplementary Material 5

Supplementary Material 6

Supplementary Material 7

Supplementary Material 8

Supplementary Material 9

## Data Availability

The datasets generated and/or analysed during the current study are available upon reasonable request. Researchers interested in accessing the data can contact the corresponding authors. Data on DEGs is provided within the supplementary information files.
