# Supplementary material for: Emerging roles of the cancerous inhibitor of protein phosphatase 2A (CIP2A) in ovarian cancer
Source: Sci Rep. 2025 Jul 1;15:22382. doi: 10.1038/s41598-025-05013-0 (PMC12214521; doi:10.1038/s41598-025-05013-0)
Supplement: Supplementary file 2 — Supplementary Material 2 [file 41598_2025_5013_MOESM2_ESM.docx]

| **miRNA** | **Betweenness** | **Input** |
| --- | --- | --- |
| hsa-miR-16-5p | 8 | 796.6562 |
| hsa-miR-26a-5p | 8 | 1029.765 |
| hsa-miR-34a-5p | 8 | 723.0616 |
| hsa-miR-182-5p | 7 | 574.1022 |
| hsa-miR-103a-3p | 7 | 431.002 |
| hsa-miR-107 | 7 | 431.002 |
| hsa-miR-205-5p | 6 | 332.6463 |
| hsa-miR-124-3p | 6 | 332.6463 |
| hsa-miR-27a-3p | 6 | 278.9435 |
| hsa-miR-17-5p | 5 | 285.2279 |
| hsa-miR-20a-5p | 5 | 379.5475 |
| hsa-miR-21-5p | 5 | 358.8483 |
| hsa-miR-93-5p | 5 | 492.4652 |
| hsa-miR-23b-3p | 5 | 230.3883 |
| hsa-miR-155-5p | 5 | 332.9704 |
| hsa-miR-106b-5p | 5 | 285.2279 |
