# Supplementary material for: Emerging roles of the cancerous inhibitor of protein phosphatase 2A (CIP2A) in ovarian cancer
Source: Sci Rep. 2025 Jul 1;15:22382. doi: 10.1038/s41598-025-05013-0 (PMC12214521; doi:10.1038/s41598-025-05013-0)
Supplement: Supplementary file 7 — Supplementary Material 7 [file 41598_2025_5013_MOESM7_ESM.docx]

**Supplementary Tables**

**Supplementary Table 1.** UK Biobank data on CIP2A

**Supplementary Table 2.** miRNA interactive network regulating the bundle of genes that were seen to be interacting with *CIP2A* based on the String analysis*.*

**Supplementary Table 3.** List of DEGs of PEO1-treated cells.

**Supplementary Table 4.** List of DEGs of PEO4-treated cells.

**Supplementary Table 5.** Patient details of the tissue microarray BC11115d

**Supplementary Table 6.** Patient details of the tissue microarray OV991
