# Supplementary material for: Emerging roles of the cancerous inhibitor of protein phosphatase 2A (CIP2A) in ovarian cancer
Source: Sci Rep. 2025 Jul 1;15:22382. doi: 10.1038/s41598-025-05013-0 (PMC12214521; doi:10.1038/s41598-025-05013-0)

Supplementary Figure 1

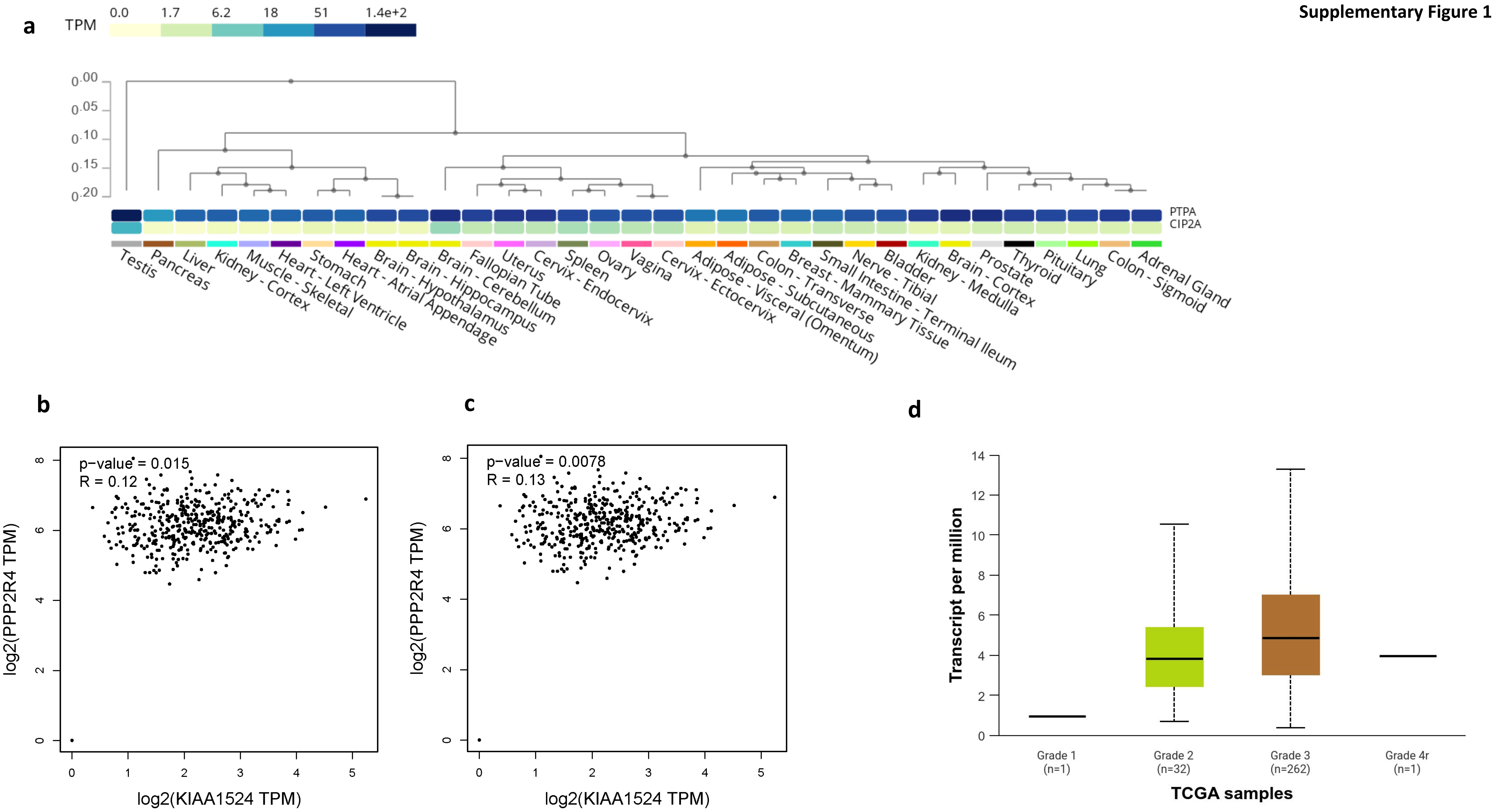

Supplementary Figure 2

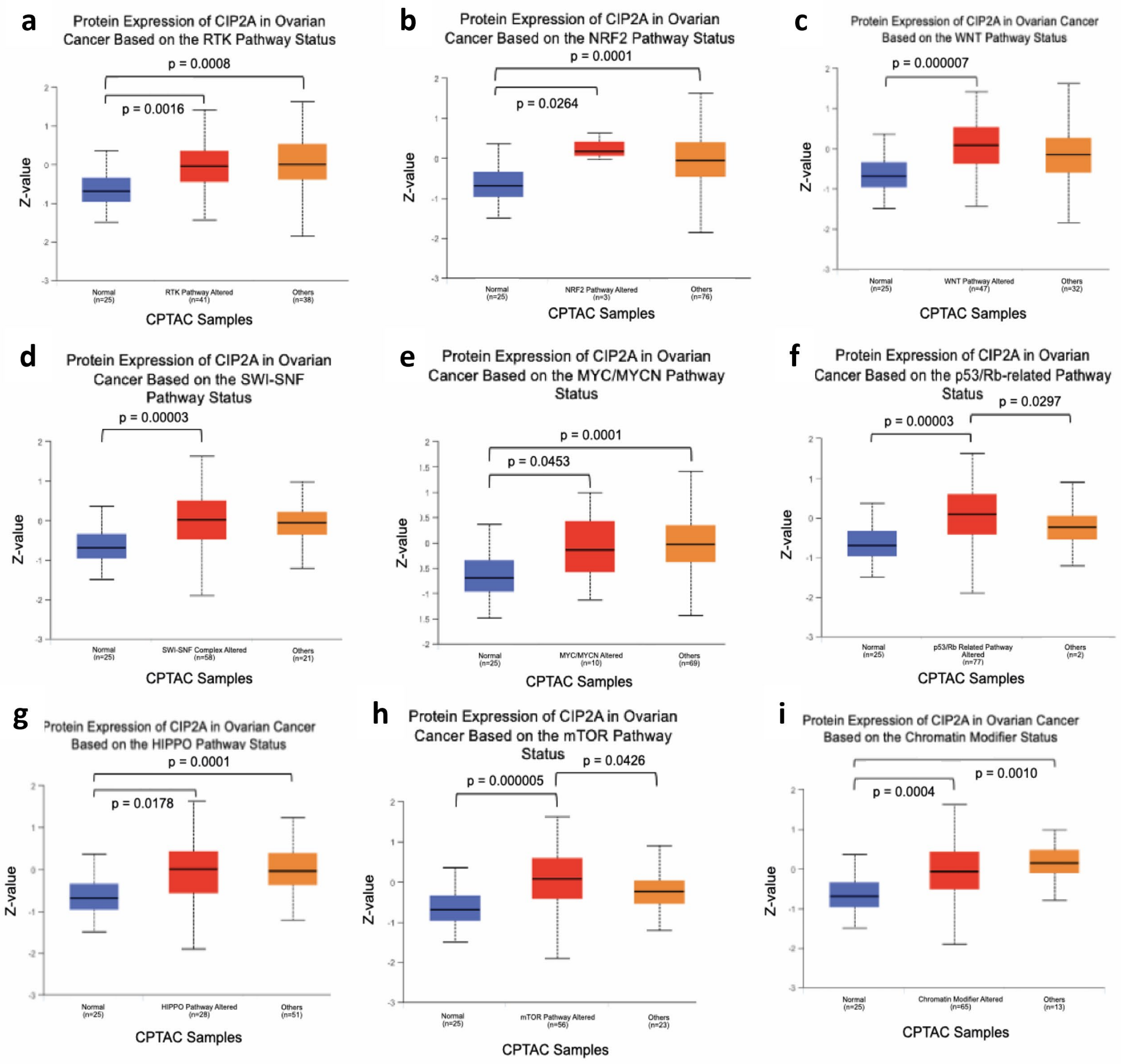

Supplementary Figure 3

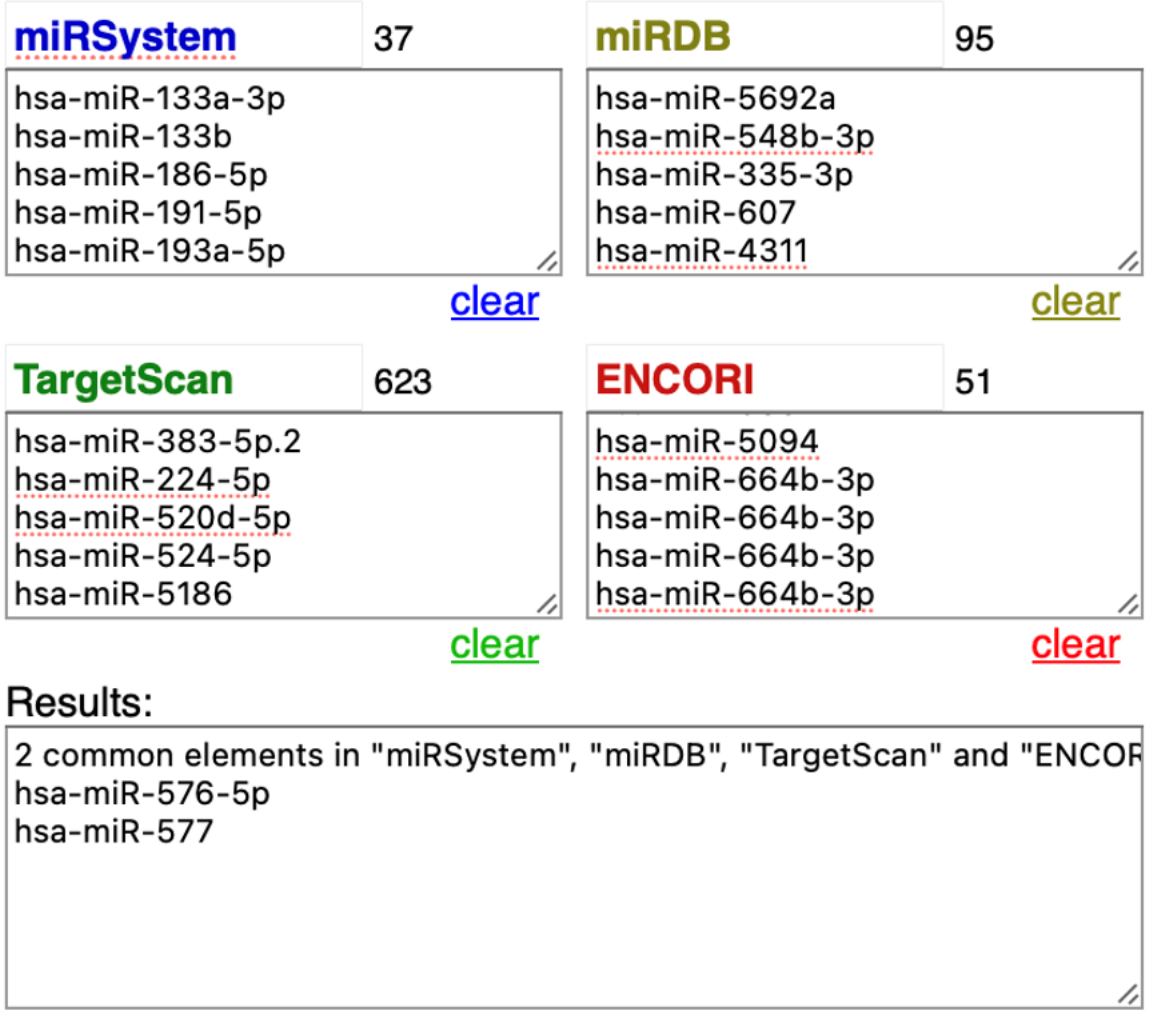

Supplementary Figure 4

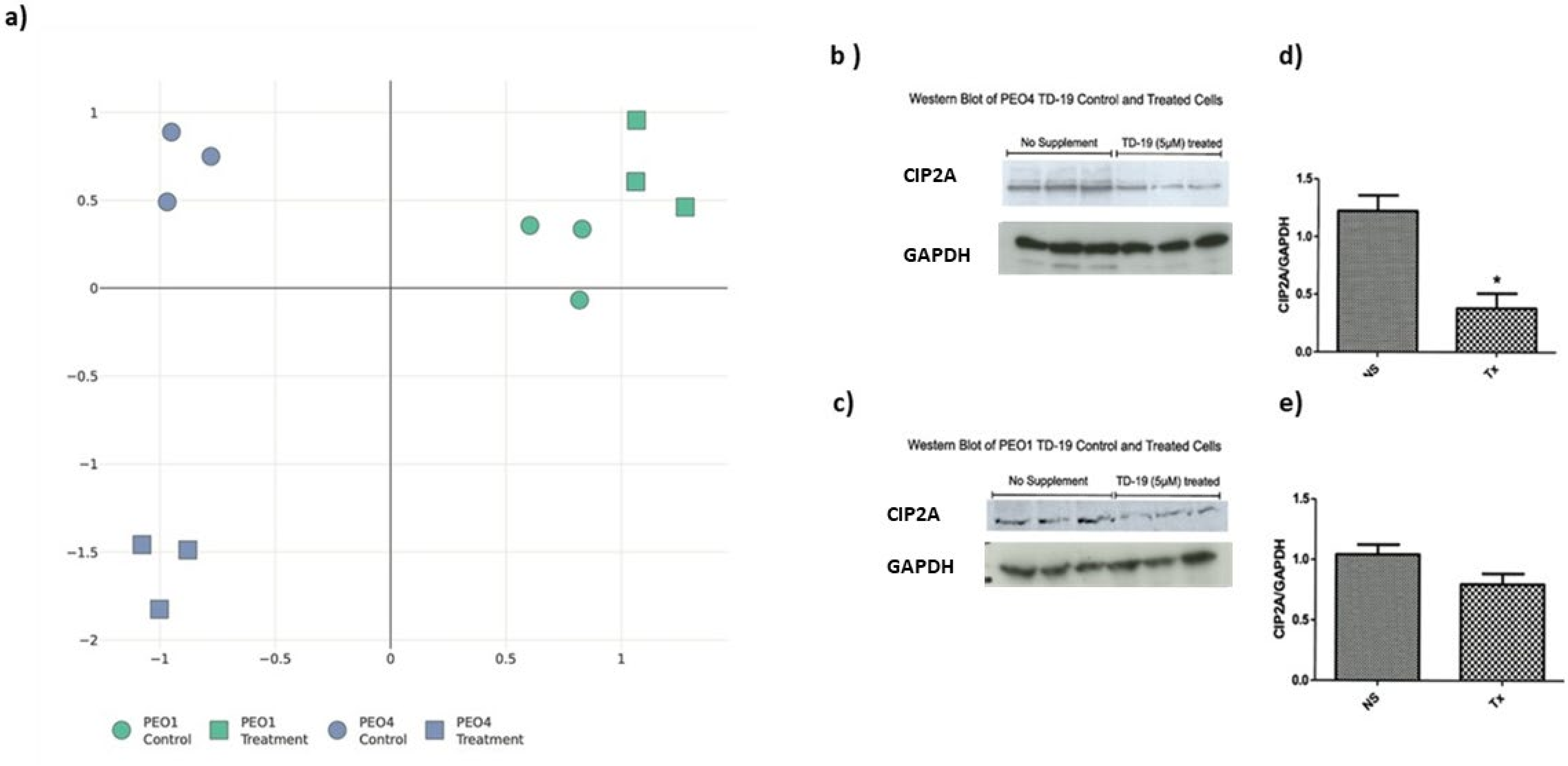

Supplementary Figure 5

PEO1

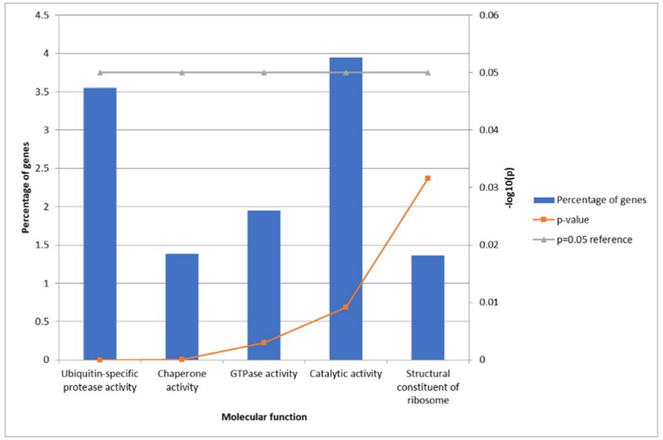

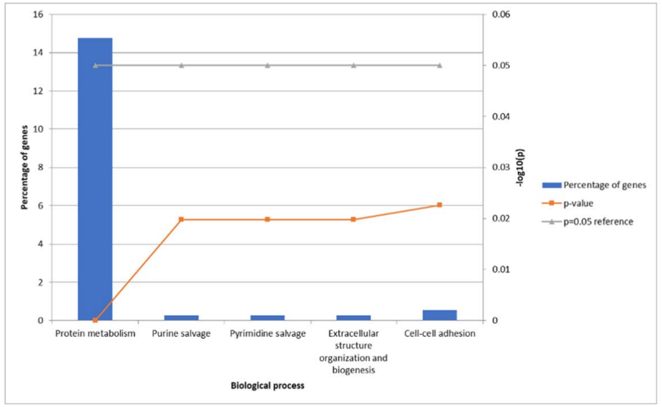

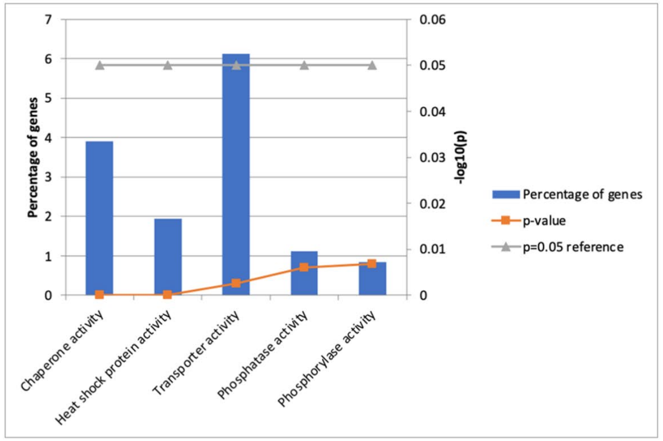

PEO4

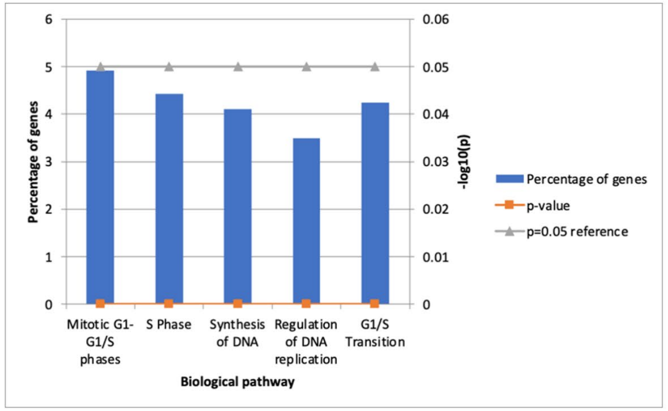

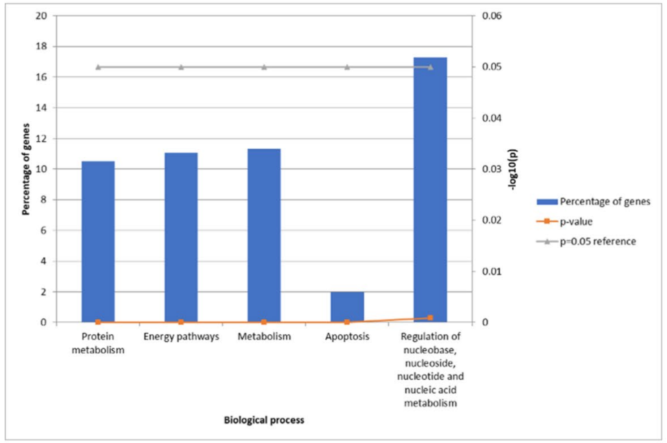

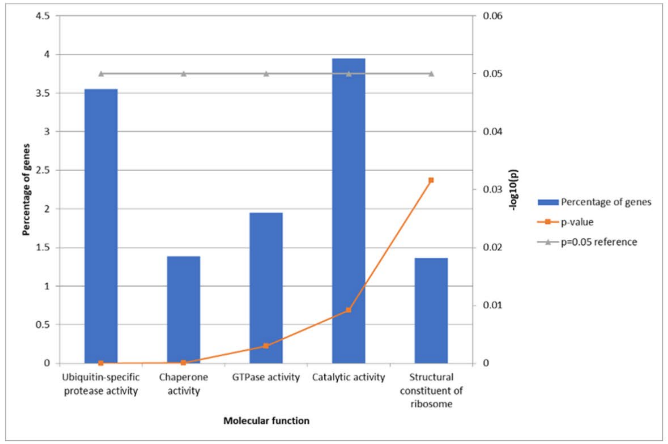

Supplementary Figure 6

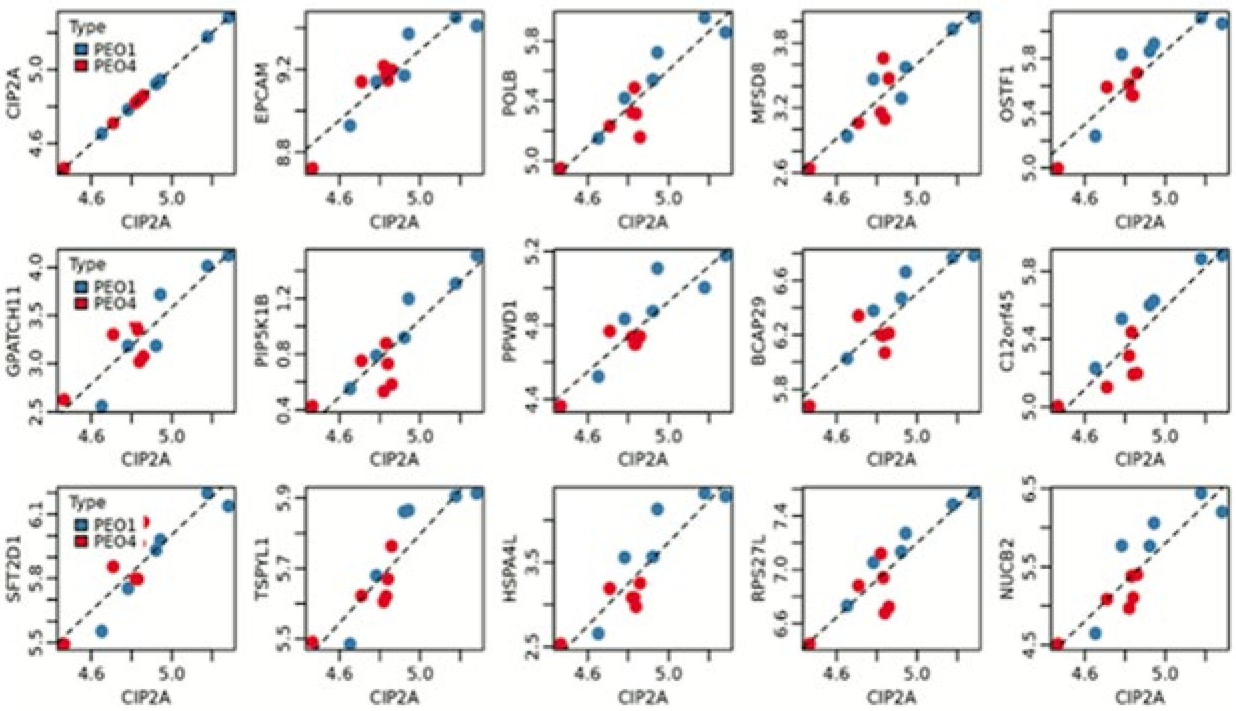

Supplementary Figure 7

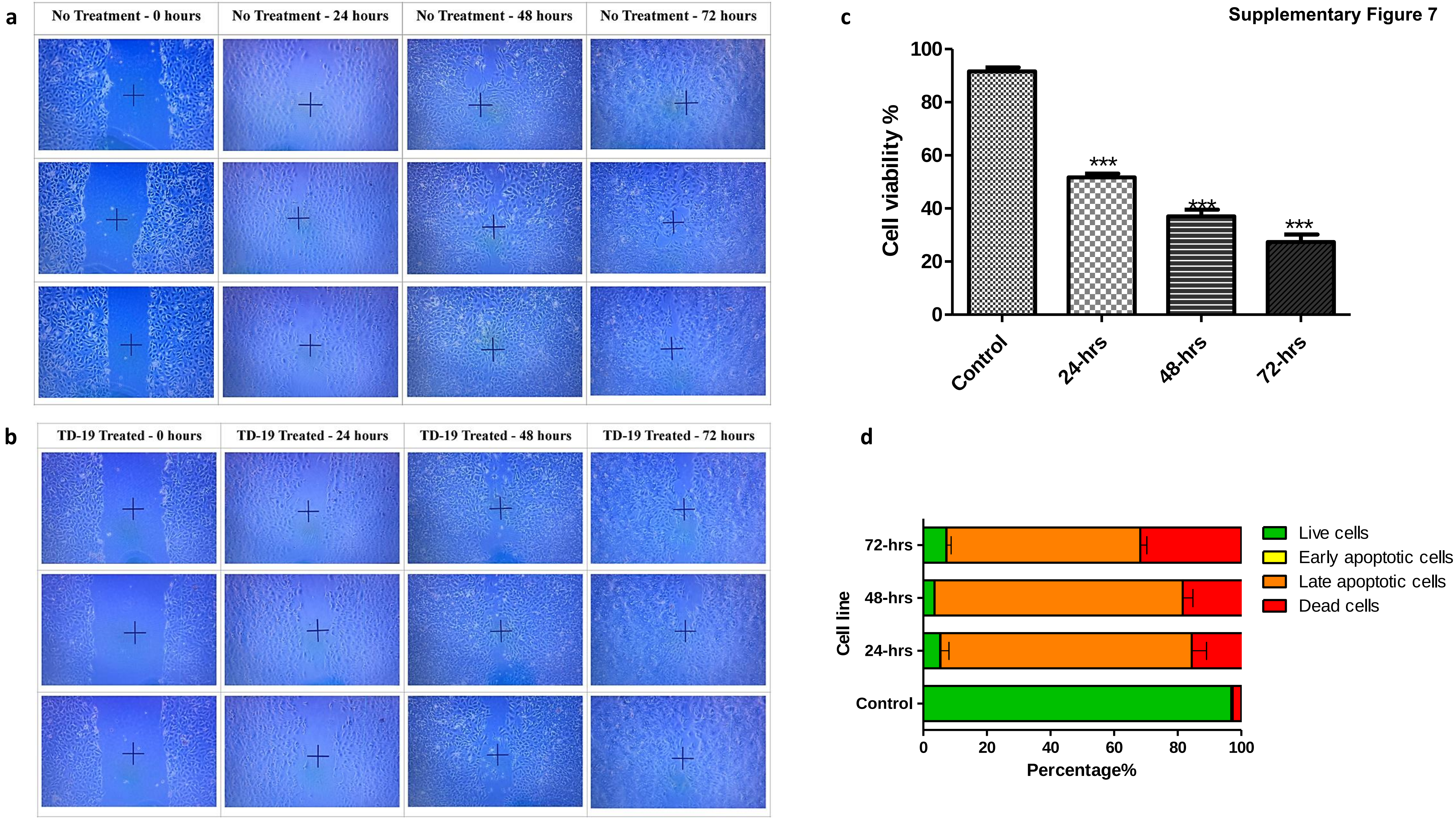

Supplementary Figure 8

a CIP2A in PEO4 cells

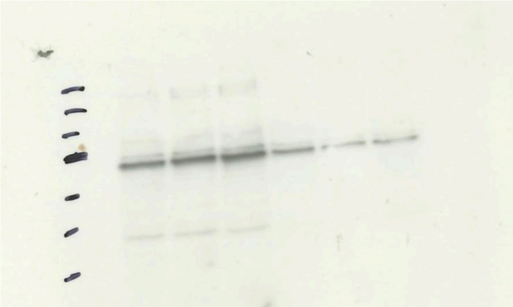

b CIP2A in PEO1 cells

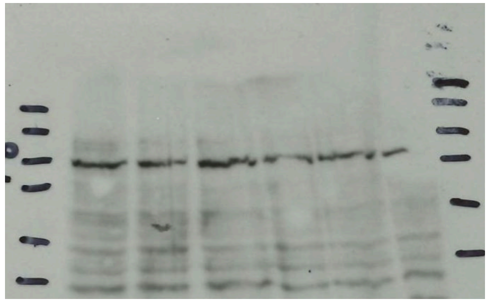

c GAPDH in PEO4 cells

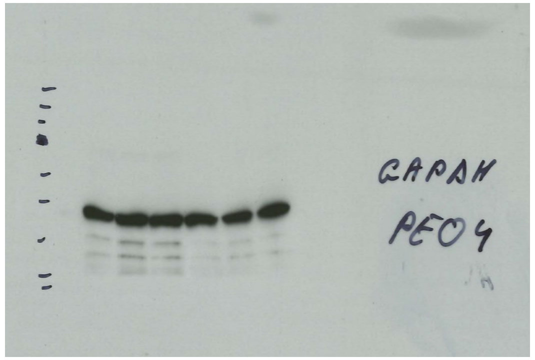

d GAPDH in PEO1 cells

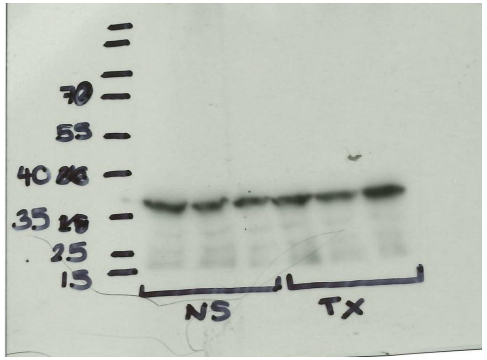

Supplement: Supplementary file 8 — Supplementary Material 8 [file 41598_2025_5013_MOESM8_ESM.pdf]
