# Supplementary material for: Emerging roles of the cancerous inhibitor of protein phosphatase 2A (CIP2A) in ovarian cancer
Source: Sci Rep. 2025 Jul 1;15:22382. doi: 10.1038/s41598-025-05013-0 (PMC12214521; doi:10.1038/s41598-025-05013-0)
Supplement: Supplementary file 9 — Supplementary Material 9 [file 41598_2025_5013_MOESM9_ESM.docx]

**Supplementary Figures**

**Supplementary Figure 1.** Co-expression of CIP2A and PP2A (PTPA) using the GTEx dataset (**a**). Weak correlation using Pearson (**b**) or Spearman (**c**) correlation analysis. *CIP2A* was upregulated in Grade 3 when compared to Grade 2 (d; p=0.05).

**Supplementary Figure 2.** Protein expression of CIP2A in OvCa patients with different signalling pathway mutations. The researched pathways include **a)** RTK, **b)** NRF2, **c)** WNT, **d)** SWI-SNF, **e)** MYC/MYCN, **f)** p53Rb-related, **g)** HIPPO, **h)** mTOR pathways, and **i**) chromatin modifier status.

**Supplementary Figure 3.** Identification of different miRNAs that can potentially interact with CIP2A.

**Supplementary Figure 4.** A t-distributed stochastic embedding (t-SNE) plot for both PEO1 and PEO4 samples (**a**; figure was generated using Omics Playground). TD-19 effect on CIP2A protein expression in PEO4 (**b**) and PEO1 cells (**c**). Densitometry analysis revealed significant downregulation of CIP2A in PEO4 (**d**) and a non-significant decrease In PEO1 (**e**). Error bars: SEM, *P<0.05.

**Supplementary Figure 5.** GO enrichment analysis for DEGs in a) PEO1 and b) PEO4 cells.

**Supplementary Figure 6.** Correlation scatter plots of *CIP2A.* Correlation scatter plots which display the expression correlation of other genes with *CIP2A* and other genes for control (blue) and treated (red) samples. All correlations noted were significant. This figure was generated using Omics Playground.

**Supplementary Figure 7**. Wound healing assay of SKOV-3 cells treated with TD-19 (5μM) from 24 to 72 hours. The gap closure was delayed in the treated cells when compared to control cells (no treatment; **a-b**). Cell viability following the same TD-19 treatment showed that it reduced cell proliferation over time, when compared to control cells (***p<0.001 comparing each treatment to control, **c**). Annexin V assay: SKOV-3 treated cells are demonstrating features of late apoptosis when compared to control (i.e. untreated cells; **d**).

**Supplementary Figure 8.** Western blot images for CIP2A expression in PEO4 (a) and PEO1 (b) cells, and GAPDH expression in PEO4 (c) and PEO1 (d) cells.
